# Fuel Cell Catalyst Layers with Platinum Nanoparticles Synthesized by Sputtering onto Liquid Substrates

**DOI:** 10.1021/acsomega.4c06245

**Published:** 2024-10-17

**Authors:** Björn Lönn, Linnéa Strandberg, Vera Roth, Mathilde Luneau, Björn Wickman

**Affiliations:** † Chemical Physics, Department of Physics, Chalmers University of Technology, Gothenburg 412 96, Sweden; ‡ Applied Chemistry, Department of Chemistry and Chemical Engineering, Chalmers University of Technology, Gothenburg 412 96, Sweden; § Competence Centre for Catalysis, Chalmers University of Technology, Gothenburg 412 96, Sweden

## Abstract

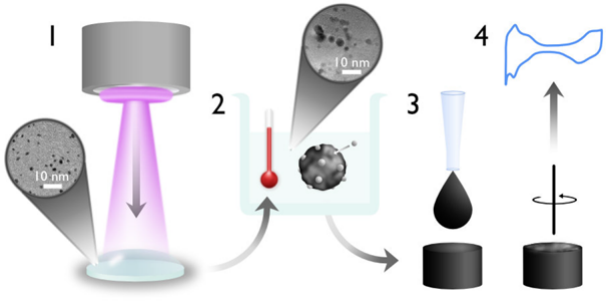

Platinum (Pt) nanoparticles are widely used as catalysts in proton exchange membrane fuel cells. In recent decades, sputter deposition onto liquid substrates has emerged as a potential alternative for nanoparticle synthesis, offering a synthesis process free of contaminant oxygen, capping agents, and chemical precursors. Here, we present a method for the synthesis of supported nanoparticles based on magnetron sputtering onto liquid poly(ethylene glycol) (PEG) combined with a heat-treatment step for attachment of nanoparticles to a carbon support. Transmission electron microscopy imaging reveals Pt nanoparticle growth during the heat-treatment process, facilitated by the carbon support and the reducing properties of PEG. Following the heat treatment, a bimodal size distribution of Pt nanoparticles is observed, with sizes of 2.5 ± 0.8 and 6.7 ± 1.8 nm, compared to 1.8 ± 0.4 nm after sputtering. Synthesized Pt nanoparticles display excellent specific and mass activities for the oxygen reduction reaction, with 1.75 mA/cm^2^_Pt_ and 0.27 A/mg_Pt_ respectively, measured at 0.9 V vs the reversible hydrogen electrode. The specific activities reported herein outperform literature values of commercial Pt/C catalysts with similar loading and are on par with values of bulk Pt and mass-selected nanoparticles of comparable size. Also, the mass activities agree well with the literature values. The results provide new insights into the growth processes of SoL-synthesized carbon-supported Pt catalyst nanoparticles, and most crucially, the high performance of the synthesized catalyst layers, along with the possibility of nanoparticle growth through a straightforward heat-treatment step at relatively low temperatures, offer a scalable new approach for producing fuel cell catalysts with more efficient material utilization and new material combinations.

## Introduction

Hydrogen fuel cells are gaining increasing global attention, driven by significant commitments from intergovernmental organizations toward a sustainable energy system, based in part on green hydrogen.^1−4^ Fuel cells have the potential to offer zero-emission energy conversion and can play a central role in a hydrogen-based energy system. The proton exchange membrane fuel cell (PEMFC) is the most widely used type of fuel cell in transportation applications, showing particular promise for long-range and heavy-duty vehicles.^5,6^ To withstand the harsh acidic environment of PEMFCs and facilitate the kinetically slow oxygen reduction reaction (ORR), platinum (Pt) is the dominating cathode catalyst material in PEMFCs, typically used in its nanoparticulate form to maximize the active surface area. The high price and scarcity of Pt, alongside its vital role within fuel cell technology, positions it as a critical metal in the coming decades.^7^ Hence, reducing the amount of Pt required in PEMFCs, for instance, by increasing its mass activity, would be highly beneficial and an important step toward a large-scale implementation of PEMFC technology.

Efforts to develop new ORR catalysts with more efficient material utilization have led to significant improvements in catalytic ORR activities, with enhancement factors of up to 10 demonstrated for Pt alloys with various late transition metals such as Ni and Co,^8−12^ as well as rare-earth metals such as Y, Gd, and Tb.^13−20^ However, a consequence of these new types of materials is the increased complexity involved in catalyst nanoparticle fabrication. Pt–rare-earth metal alloys constitute an illustrative example, as the high oxygen affinity of rare-earth metals prevents nanoparticle synthesis with traditional wet chemical methods.^21^ Hence, while the development of highly active catalyst materials is crucial, it is also essential to develop new scalable fabrication techniques compatible with the increasing demand and complexity of these novel catalysts.

One of the few techniques that have been successfully employed in the fabrication of Pt–rare-earth metal alloy nanoparticles so far is sputtering.^14,15^ In fact, sputtering is a very powerful synthesis technique, given its large range of compatible materials,^22^ making it a suitable candidate for synthesizing a wide variety of nanoparticles. However, for the fabrication of real fuel cell catalysts, traditional sputtering onto high surface area carbon supports does not allow the dispersion of catalyst particles over the full active electrode volume. Furthermore, the mass selection needed to create size-controlled nanoparticles in conventional sputtering is not compatible with mass production.^23^

When sputtering on a liquid substrate rather than solid supports, the method presents a fabrication pathway that could potentially overcome the issues related to scalability and material dispersion. The principle of sputtering onto liquid substrates is that a low vapor pressure liquid is used as a medium for both the collection and growth of the sputtered material. This allows for nanoparticles to be produced, collected, and stored in the liquid, and later to be transferred to a suitable support, expanding the applicability of sputtering fabrication significantly. Since first introduced by Ye et al. in 1996^24^ as a new means of sputtering thin films, the development of the method has moved toward nanoparticle synthesis. A variety of single metals, including Au,^25−28^ Ag,^29^ Cu,^30^ and Pt,^31−37^ alongside a plethora of metal alloys,^38−40^ have been synthesized by sputtering onto liquids. In the case of Pt nanoparticles, the main body of work has focused on the employment of various ionic liquids,^33,35−37^ polymer poly(ethylene glycol) (PEG),^32−34^ or glycerol,^31^ as sputtering substrates. While Pt particles produced in glycerol typically suffer from extensive aggregate formation,^31^ ionic liquids produce small Pt nanoparticles, around 1–2 nm in diameter and with high stability against aggregation,^33,35−37^ through the formation of an ionic double layer around the particles. While ionic liquids constitute a vast group of materials, some of which could be interesting liquid substrate alternatives, their environmental impact and toxicological effects are yet to become fully established.^41^ In addition, ionic liquids are generally expensive compared to traditional organic solvents.^42^ For this study, PEG was chosen as a sputtering substrate, as it is a nontoxic polymer, widely used as a food and pharmaceutical additive, and with great prospects as a green solvent.^43^ Furthermore, PEG combines the advantages of glycerol and ionic liquids, offering both efficient nanoparticle stabilization and low cost, making it a suitable liquid substrate for nanoparticle synthesis.

For real fuel cell applications, catalyst nanoparticles are typically attached to a high surface area support material, in order to provide electrical conductivity, chemical stability, and efficient transport of reactants and products and facilitate a large active surface area. Hence, the attachment of catalyst particles to the carbon support material is an important process step in the fabrication of practical catalysts. Cha et al.^34^ reported a simple one-step synthesis of 2 nm diameter Pt nanoparticles on carbon support, based on radio frequency (RF) sputtering onto PEG. These particles are, however, slightly too small compared to the reported optimum size of around 3 nm in diameter^44^ for Pt ORR catalyst nanoparticles, which is reflected in their observed ORR activity. In fact, little information revolving around the tuning of Pt nanoparticle size when sputtered into PEG exists in the literature, especially for the case of supported nanoparticle catalysts for ORR. Yet, size effects are profoundly important when investigating potential synthesis routes for catalyst material. A better understanding of nanoparticle growth processes, both during sputtering and during attachment to support materials, is of great importance to establish sputtering onto liquids as a viable synthesis route for PEMFC catalysts. This is particularly true if the technique is to be expanded to include other types of materials, which exhibit much larger particle sizes for optimum ORR activity, as is the case for Pt-RE alloys.^14,15^

In the present study, direct current (DC) magnetron sputtering of Pt onto PEG is combined with a heat-treatment process at 150 °C for nanoparticle production and subsequent transfer from liquid substrate to a Vulcan XC 72 carbon support. Differently from the work by Cha et al.,^34^ this combined method allows further growth of the supported Pt nanoparticles beyond 2 nm in diameter, as revealed by X-ray diffraction (XRD) measurements, and transmission electron microscopy (TEM) imaging before and after attachment to the support material. Significant growth of the catalyst particles occurs during the heat treatment, from a single population of primary Pt particles of 1.8 nm to a bimodal distribution of 2.5 and 6.7 nm particles. This growth is attributed to the presence and participation of carbon support within the reducing PEG environment. In addition, the larger Pt particle size in this work results in higher specific and mass ORR activities, compared to the particles synthesized by Cha,^34^ and compared to literature values of commercial Pt catalysts studied using the same experimental conditions,^45^ the particles exhibit higher specific activities and comparable mass activities. Our results showcase a simple and efficient particle-to-catalyst ink pathway, with the possibility to further grow supported catalyst nanoparticles postsputtering, an ability that is of utmost importance when optimizing the size of Pt-based ORR catalyst nanoparticles, and now demonstrated for the case of the SoL technique. Most essentially, it illustrates the potential of sputtering onto PEG as an alternative ORR catalyst fabrication technique and could potentially have interesting applications for more complex Pt-based systems, not compatible with conventional synthesis methods.

## Experimental Section

### Supported Nanocatalyst Fabrication

**Figure 1:**
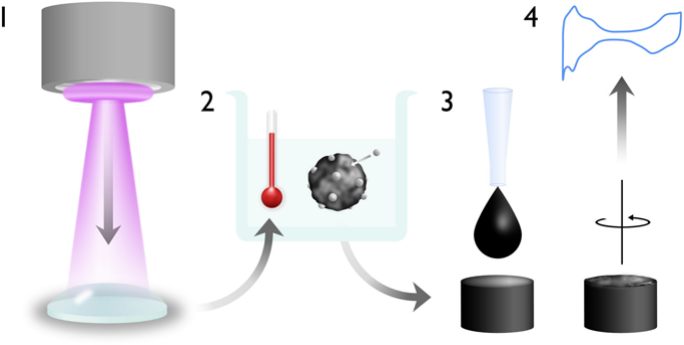
Schematic illustration of the individual process steps: magnetron sputtering onto PEG (1), heat-treatment for nanoparticle attachment to carbon support (2), ink preparation and drop casting (3), and electrochemical characterization (4).

A schematic of the nanocatalyst synthesis steps is shown in Figure 1. Dispersed, liquid-stabilized Pt nanoparticles (from here on referred to as Pt primary particles) were synthesized by means of DC magnetron sputtering onto PEG 600 (Thermo Fisher Scientific), using a custom-built sputter coater.^33^ The liquid substrate, placed on top of a fused silica glass wafer (Mark Optics), was mounted beneath the magnetron gun, equipped with a Pt target (99.99% pure from Kurt J. Lesker) and with a target-to-substrate distance of 8 cm. The chamber was then pumped to ultrahigh vacuum (UHV), to a base pressure of less than 5 × 10^–7^ mbar. For the sputtering, a constant flow of 30 sccm argon (Ar) (Argon 6.0 Strandmo̷llen) was introduced to the chamber, reaching a working pressure of 9 × 10^–3^ mbar. A magnetron power of 50 W was used, resulting in sputtering voltages and currents of 415 ± 7 V and 121 ± 3 mA, respectively, and samples were sputtered for three 300 s cycles with 20 min of cooling between each cycle.

The Pt primary particles in PEG were transferred to a carbon support via a heat-treatment step. First, 2.5 mg of Vulcan XC 72 was added to 185 μL of Pt/PEG solution and dispersed by ultrasonication in an ice bath for 30 min. The obtained slurry was heat-treated at 150 °C in an oil bath, equipped with magnetic stirring (100 rpm), for 19 h. Following the heat treatment, a supported nanocatalyst powder was acquired through a cleaning step involving dispersion in 2-propanol (IPA) and powder extraction by centrifugation. The cleaning process was repeated three times to achieve adequate PEG removal, after which the collected wet powder was dried in an oven at 80 °C overnight.

A catalyst ink, used to coat glassy carbon electrodes (GCEs) for rotating disk electrode (RDE) measurements, was prepared by first dispersing 1.0 mg of catalyst powder in 2.0 mL of a solvent mixture (67 vol % IPA, 33 vol % 18.2 MΩ•cm Milli-Q water) through ultrasonication in an ice bath for 20 min. A small amount of Nafion 117 solution (1100 equiv weight, 5 vol %, Sigma-Aldrich) was added to the ink mixture, followed by additional sonication for 10 min. The catalyst ink was then drop cast (10 μL) onto the GCEs and dried using a rotating holder, spinning at 20 rpm, combined with convective heating applied by a heat gun. The drop-casting procedure was performed twice for each sample.

### Electrochemical Characterization

An RDE setup (Pine Research), connected to a potentiostat (SP-300, Biologic), was used for the electrochemical characterization. A glass cell was cleaned using piranha solution and filled with 0.1 M HClO_4_ (Perchloric acid 70% Suprapur, Merck, diluted to 0.1 M using 18.2 MΩ•cm Milli-Q water) electrolyte. An electrolyte pH of 1.0 was confirmed by a pH meter (FiveEasy F20 equipped with an LE438 sensor from Mettler Toledo). The cell was then equipped with a Hg/Hg_2_SO_4_ reference electrode (SI Analytics) alongside two Pt wires, one used as a counter electrode during measurements and the other for potential control during sample immersion. The electrolyte was saturated by bubbling of Ar (Argon 6.0, Strandmo̷llen) for 20 min before any experiments were conducted. For estimation of the ohmic resistance of the setup, electrochemical impedance spectroscopy (EIS) was performed by scanning the frequency between 20,000 and 10 Hz and plotting the impedance in a Nyquist plot. The ohmic drop of the system was then estimated as the intersection between the extrapolated impedance and the real impedance axis and used to IR-compensate measured data.

This study uses the reversible hydrogen electrode (RHE) as a common reference for all potentials reported herein. To determine the potential of the reference electrode relative to RHE, the potential was cycled between −0.7 and −0.74 V vs the Hg/Hg_2_SO_4_ reference electrode, in hydrogen-saturated (Hydrogen 6.0, Strandmo̷llen) electrolyte, at a rotation of 1600 rpm and using a scan rate of 1 mV/s. With RHE determined as the average intercept with the voltage axis (zero current), the reference electrode potential was found to be 0.720 V vs RHE.

Cyclic voltammetry (CV) was performed in both Ar-saturated and oxygen-saturated (Oxygen 5.2, Strandmo̷llen) electrolytes for background and oxygen reduction CVs, respectively, by cycling the potential between 0.05 and 1.0 V vs RHE at 50 mV/s. In the case of oxygen reduction, a rotation rate of 1600 rpm was used to improve the mass transport of oxygen to the electrode. The electrochemical surface area (ECSA) was determined by CO stripping, in which the sample was held at a constant potential of 0.05 V vs RHE for 20 min, while its surface was intentionally poisoned by CO bubbling (10% CO (3.7) in Ar (6.0), Strandmo̷llen) for three min followed by purging of the excess CO by Ar bubbling for 17 min. Then, the potential was cycled between 0.05–1.0 V vs RHE at 10 mV/s scan rate, and the CO oxidation peak charge was obtained by subtraction of the following cycle and integration of the resulting peak. By comparing this charge to the specific charge of 420 μC/cm^2^_Pt_, generally assumed to correspond to a monolayer of adsorbed CO on polycrystalline Pt,^46,47^ the ECSA can be obtained.

EC-Lab software was used to correct all data for 85% of the ohmic resistance during the measurement, while a manual correction was implemented for the remaining 15% during the data analysis. Finally, the ORR data was corrected by subtracting the Ar background CV and normalized by the ECSA. Kinetic current densities (*j*_K_) were extracted utilizing the Koutecký–Levich equation:
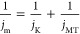
where *j*_m_ and *j*_MT_ are the measured and mass transport limited current densities, respectively.

### Physical Characterization

Nanocatalyst size and morphology were evaluated by transmission electron microscopy (TEM) imaging. For supported catalyst samples, the catalyst powder was initially dispersed in IPA, drop cast on Au lacey carbon TEM-grids (Ted Pella, Inc., 300 mesh), and left to dry. Primary Pt particles in PEG were transferred to TEM grids by casting a drop on the grid and leaving it for 4 h, to allow attachment of nanoparticles to the TEM grid. Excess Pt/PEG solution was removed by dropwise cleaning with acetonitrile (VWR) on the opposite side of the grid to prevent the removal of attached particles. Imaging was performed in an FEI Tecnai T20, operating at 200 kV acceleration voltage, or an FEI Titan operating with 300 kV acceleration voltage. Size distributions of catalyst nanoparticles were obtained from TEM micrographs by hand measurements in the software ImageJ, of over 150 particles per sample. For the supported catalyst sample, only clearly distinguishable particles were counted, resulting in larger aggregates not being considered.

XRD patterns were acquired between 15 and 80° 2Theta on a Bruker D8 Discover diffractometer in a Theta-2Theta configuration, equipped with a Cu tube X-ray source and an Eiger2 R 500 K 2D detector set to 1D mode. The sample was ground to a fine powder and placed on a zero-background Si single crystal holder. Data acquisition time was set to 2.5 h, at 0.02° increment. The divergence slit on the incoming beam was set to fixed sample illumination of 8 mm, and an air scattering shield was used in automatic mode. 2.5° soller slits were inserted on the primary and secondary sides. Instrumental broadening was derived from a Corundum standard measurement by using the same scan parameters. Rietveld refinement was performed using TOPAS v6. Pearson VII functions were used for simulating the peaks. Chebychev polynomial with four parameters was used for simulating the background. We applied zero error correction and used two Pt Cubic *Fm*3̅*m* structures (*a* = 3.92 Å) to fit the data set and obtained an *R*_wp_ value of 0.97%. Peak intensity variations were treated using spherical harmonics with eight parameters. The data set is shown in Figure 3, and the fit in Figure SI7.

Chemical composition of the surface species was investigated with X-ray photoelectron spectroscopy (XPS) with a PHI 5000 VersaProbe III spectrometer equipped with a monochromatic Al Kα source. Scan measurements were aligned with the C-C bond (C 1s) centered at 284.4 eV as described in a previous XPS study performed on Vulcan XC 72.^48^

To determine the Pt loading on the electrodes, inductively coupled plasma mass spectrometry (ICP-MS) was used. A small amount of catalyst powder (1.17 mg) was dissolved in 1 mL of aqua regia (HCl 30% Suprapur, Merck and HNO_3_ 69%, Suprapur, Merck, with a molar ratio of 3:1), and subsequently diluted with 0.5 M HNO_3_ (69%, Suprapur, Merck, diluted to 0.5 M using 18.2 MΩ•cm Milli-Q water). In addition, 2 μg/L Bi (bismuth, 10 mg/L in 2% HNO_3_, VHG Labs) was used as the internal standard for ICP-MS analysis.

## Results and Discussion

### Nanocatalyst Morphology and Composition

**Figure 2:**
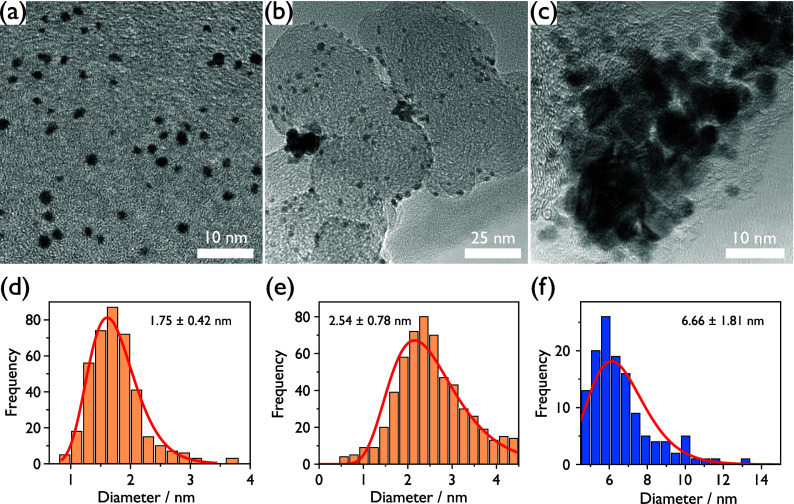
Attachment of Pt nanoparticles onto carbon via heat-treatment results in larger particle sizes, as shown by TEM micrographs of Pt nanoparticles sputtered in PEG (a) before and (b, c) after attachment to carbon support via a heat treatment step. The bottom row displays resulting particle size distributions (d) before and (e, f) after the heat-treatment, highlighting the dual nanoparticle populations present after the heat treatment. Additional images used for particle counting are included in Figures SI4–SI6 of the Supporting Information.

Sputtering Pt onto PEG 600 results in well-defined small nanoparticles with a narrow size distribution (Figure 2a,d). TEM images acquired after sputtering show that primary nanoparticles sputtered onto PEG 600 have a diameter of around 1.8 nm. The nanoparticles are well dispersed under the steric stabilization provided by the substrate.

Attachment of Pt nanoparticles to high surface area carbon and heat treatment result in two populations of particles (Figure 2b,c). The first, which is present in both pictures but highlighted in Figure 2b, is that of primary particles, forming a well-dispersed decoration across the carbon support. These particles are of comparable size to the as-sputtered primary particles, exhibiting a slight increase in mean diameter from 1.8 to 2.5 nm (Figure 2e). This increase in the mean size of primary nanoparticles is larger than previously reported,^33^ which could potentially be explained by the longer heat treatment performed in the present work. XPS Pt 4f narrow scans (Figure SI9 of the Supporting Information) indicate that the surface of the heat-treated Pt nanoparticle is partly oxidized, although this oxide is not present after the ORR experiments.

**Figure 3:**
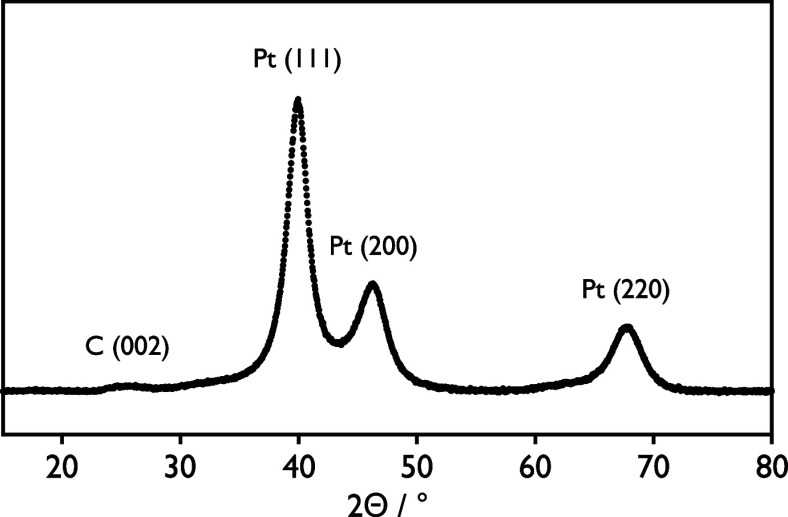
XRD pattern of heat-treated Pt nanoparticles on Vulcan XC 72.

Additionally, the presence of a carbon support has an apparent impact on the continued growth of sputtered Pt nanoparticles, which becomes evident when considering the second population (Figure 2c,f). This population, consisting of considerably larger nanoparticles (6.7 nm mean diameter), mostly appears in, or in close vicinity to, large aggregates. Consequently, extracting a size distribution of clearly distinguishable particles was more difficult for this population, and thus the histograms most likely underestimate the relative fraction of this larger particle population. Albeit only considering the indicated sizes of the second particle population as a rough estimate, it is clear that these particles are markedly larger than the primary particles. Complementary XRD-measurement of the heat-treated nanocatalyst powder is presented in Figure 3 and displays diffraction peaks at 2θ angles of 39.9, 46.3, and 67.8°, corresponding to the Pt (111), Pt (200), and Pt (220) face-centered cubic reflections, respectively. The smaller peak observed between 23.5 and 27.5° corresponds to the C (002) Bragg peak originating from the hexagonal graphite structure of Vulcan XC 72. Overall, the pattern is very similar to existing reports on Vulcan-supported Pt nanoparticles.^49^ Fitting the obtained XRD pattern (see Figure SI7 and corresponding section of the Supporting Information) required the use of two Pt phases to obtain good agreement with the experimental data and predicts two crystallite sizes of 2.4 and 4.6 nm, respectively.

While XRD does not provide a complete description of the full particle size distribution, it clearly demonstrates crystallite and nanoparticle growth far beyond 2.5 nm, supporting the trend observed in TEM. Such a dramatic size increase is unlikely to have been induced by only heating, based on the minor influence seen in our previous study,^33^ leaving only the presence of carbon particles to explain this behavior. Carbon particles have been shown to impact the size of Pt-based nanoparticles in previous work by Campos-Roldán et al.^50^ Further, Sellin et al.^51^ studied the influence of both temperature and atmosphere on carbon-supported Pt nanoparticles. Their results indicated varying degrees of particle agglomeration without crystallite growth, during heat-treatment in inert atmosphere (Ar) and air, for temperatures up to 523 K. However, with the introduction of a reducing atmosphere (3% H_2_ in He), significant growth was observed, even at temperatures as low as 373 K. The reducing properties of PEGs have been demonstrated and utilized in Pt nanoparticle synthesis^52^ and hence could provide the reducing environment needed for nanoparticle growth.

**Figure 4:**
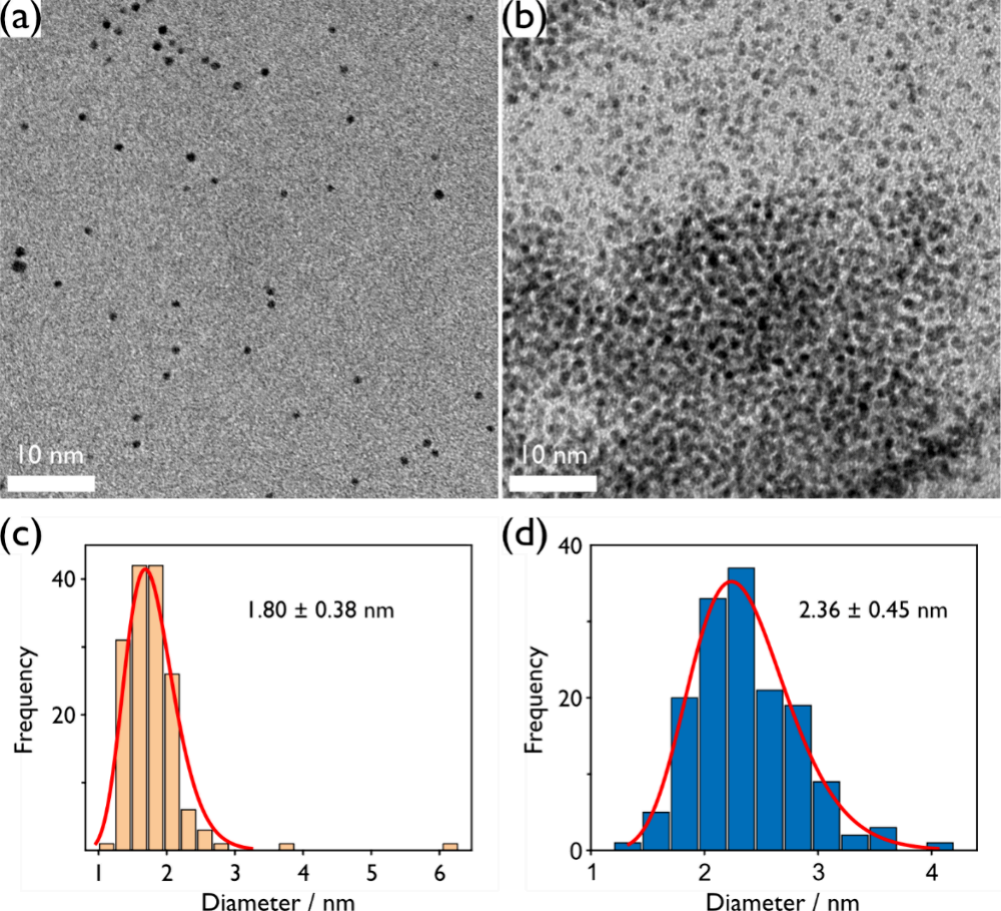
TEM micrographs of heat-treated primary particles. Heat treatment of Pt nanoparticles in the absence of a carbon support results in partial separation of the immersed primary particles by aggregation. Particles remaining in solution are well dispersed and of small size as shown in (a) and (c), while separated particles exhibit slightly larger particles, with a large degree of aggregation, displayed in (b) and (d).

To confirm that the increased Pt particle size observed herein is limited to supported Pt particles, heat treatment was performed on the as sputtered primary particles in PEG. In the absence of the support material, the results show a partial separation of primary particles via aggregation. Particles that are still immersed in the liquid substrate after heat treatment are well dispersed with small diameters and a narrow size distribution (Figure 4a,c). Separated particles show a large degree of agglomeration and aggregation with slightly larger particles (Figure 4b,d). However, neither of the two populations displays particle growth past 2.5 nm in diameter. Hence, the further growth of Pt nanoparticles to 6.7 nm in mean diameter shown for the second population of the supported sample, is attributed to the presence of carbon support during the heat-treatment step in combination with the reducing environment provided by PEG, although the exact role of the support is not clear at this time. It is important to note that a majority of the catalyst material and surface area belongs to the population of larger-sized nanoparticles, which is expected to be reflected in the specific ORR activity.

### Oxygen Reduction Performance

**Figure 5:**
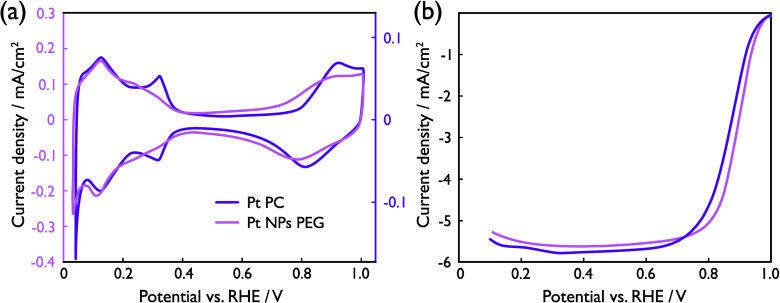
Cyclic voltammograms of supported Pt nanoparticles and polycrystalline Pt. The heat-treated, carbon supported Pt nanoparticles (light pink) show (a) characteristic adsorption and desorption regions in Ar-saturated 0.1 M HClO_4_ and (b) typical ORR activity in oxygen-saturated 0.1 M HClO_4_, as shown by geometric current densities. Polycrystalline Pt (dark pink) is shown for reference. Potentials were cycled between 0.05 and 1.0 V vs RHE, at a scan rate of 50 mV/s at 22 °C, and currents were normalized by the electrode area. A rotation rate of 1600 rpm was used for the ORR measurement in (b).

**Figure 6:**
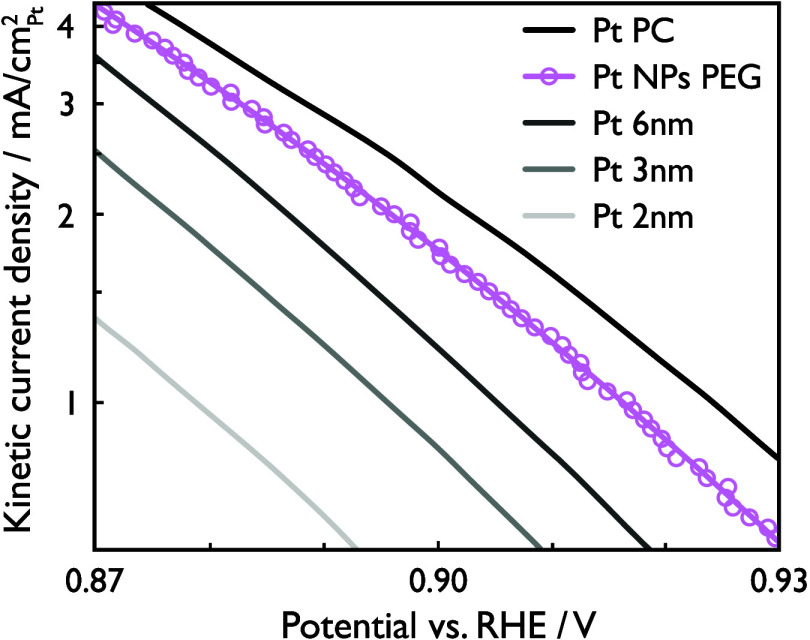
Supported Pt nanoparticles present high ORR activity, as shown by kinetic current densities of supported Pt nanoparticles sputtered in PEG (this work, light pink), polycrystalline Pt (black), and mass-selected nanoparticles between 2 and 6 nm (gray scale). Polycrystalline bulk and mass-selected particle activities are shown for comparison and were retrieved from Perez-Alonso et al.^44^

The presence of Pt nanoparticles in the drop casted catalyst layer is confirmed with electrochemical measurements in Ar (Figure 5a). The Ar CV depicts hydrogen and oxygen adsorption–desorption regions characteristic of Pt nanoparticles in acidic media. Furthermore, the capacitive contribution from the carbon support is clearly seen in the capacitance double-layer region. Overall, the Ar CV resembles the existing literature of supported Pt nanoparticles,^53^ suggesting the successful application of the catalyst layer onto the GCE. A deeper analysis of the deposited catalyst layer, in the form of CO-stripping and ICP-MS measurements, reveals ECSA and Pt mass loadings of 0.46 cm^2^ (15.6 m^2^/g_Pt_) and 15 μg_Pt_/cm^2^, respectively. The geometric ORR activity features an onset potential close to 1.0 V, and a limiting current of −5.6 mA/cm^2^ (Figure 5b), which is within 10% of the theoretical value of −6.0 mA/cm^2^_disk_.^53^ Kinetic current densities (Figure 6) were calculated from the ORR data through normalization by the ECSA, and correction for mass transport limitations via the Koutecký-Levich equation (see the Supporting Information for more details on the ORR analysis). In addition to the supported Pt nanoparticles obtained in this work, kinetic current densities of polycrystalline Pt and mass-selected nanoparticles with diameters ranging between 2 and 6 nm, originally reported by Perez-Alonso et al.,^44^ are included for comparison purposes. Looking at these specific activities, the supported Pt particles sputtered in PEG exhibit high catalytic activity, placed between those of mass-selected 6 nm Pt particles and polycrystalline bulk Pt, and considerably higher than those of 2–3 nm Pt particles. This confirms the indications seen in TEM and XRD, that the catalytically active surface is dominated by the larger particle population. Furthermore, the high specific activity indicates that PEG was efficiently removed during the washing step.

For a meaningful analysis of catalyst performance, specific and mass ORR activities of the heat-treated carbon-supported Pt nanoparticles are compared to both the mass-selected model catalyst particles,^44^ and commercial Pt/C catalyst powders,^45^ in Table 1. The SoL synthesized particles display a significantly lower ECSA compared to the commercial Pt/C catalysts, which is likely a consequence of the larger degree of aggregation seen for the samples in the present study based on the TEM analysis. Nonetheless, the specific activity is considerably higher than for mass-selected 2 and 6 nm particles as well as the commercial powders, which can be explained by the larger particle size in the present work. Additionally, when compared with commercial catalysts, the fact that the SoL synthesis method does not rely on chemical precursors or surfactants, or any harsh technique for removal of the latter,^54^ most likely plays a role in the higher specific activities observed here. Moreover, the mass activity is comparable to those of the commercial catalysts, while the lower mass activity compared to mass-selected nanoparticles can be explained by the more idealized conditions (better oxygen diffusion due to the lack of support material) in that study.^44^ Overall, the SoL synthesized Pt/C nanocatalyst displays excellent ORR catalytic performance, despite a lower ECSA than in the compared reports, indicating that the mass activity could possibly be improved by decreasing the nanoparticle aggregation and hence obtaining higher values for the ECSA.

**Table 1:** ECSA, Particle Diameter, Pt Loading, Specific and Mass Activities of Heat-Treated Carbon-Supported Pt Nanoparticles (this Work), Mass-Selected Pt Nanoparticles,^44^ and Commercial Pt/C Catalysts with Varying Pt Contents^45^^a^

sample	ECSA (m^2^/g_Pt_)	particle size (nm)	Pt loading (μg_Pt_/cm^2^)	specific activity (mA/cm^2^_Pt_) @ 0.9 V vs RHE	mass activity (A/mg_Pt_) @ 0.9 V vs RHE
SoL Pt/C, heat-treated (this work)	15.6	bimodal 2.5 and 6.7	15	1.75	0.27
mass-selected 2/6 nm particles ref (44)		2/6		0.37/1.2	0.53/0.58
commercial Pt/C catalysts 20.1/46.6/50.6 wt % Pt ref (45)	108/76/46	2/2–3/4–5	14/14/14	0.51/0.49/0.50	0.548/0.374/0.227

a All activities were measured by using a scan rate of 50 mV/s and 1600 rpm rotation speed.

### Applicability of the Technique

The results obtained here showcase the efficient synthesis of high-performance catalyst layers by combining sputtering onto a liquid with subsequent heat treatment of the suspension mixed with a high surface area carbon support. Scalability of the sputtering onto liquid technique has previously been demonstrated by Meischein and Ludwig^55^ for Cu particles in the imidazolium-based ionic liquid Bmim Tf_2_N. Their findings showed only minor changes in nanoparticle properties when increasing the process yield by 4 orders of magnitude. Furthermore, the nanoparticle output per unit time in Meischein’s work falls between that of ball milling and chemical synthesis, which are two techniques commonly used for large-scale synthesis. An extension of the method to produce the supported nanoparticles reported here requires only the addition of support material and a heating step following the sputtering, indicating the potential for scale-up for the technique presented in this work. However, further investigations are needed to confirm this.

The larger nanoparticle sizes obtained in this work compared to previously reported Pt particles sputtered onto liquids could be of interest for catalyst synthesis involving materials such as the Pt-RE metal alloys, as they require larger nanoparticle sizes than pure Pt to provide ORR activity enhancements and achieve the optimal material utilization.^14,15^ However, several aspects still need to be investigated for such a fabrication route. For example, it would require heat treatment in vacuo, which might in turn affect the nanoparticle growth process.

Moreover, it would be of interest to study ways to reduce the degree of nanoparticle aggregation as the aggregation likely limits the mass activity. For this reason, to implement the strategy employed by Cha et al.,^34^ sputtering directly onto carbon support particles dispersed in PEG could be beneficial, as they observe less aggregation of Pt nanoparticles. Additionally, the temperature and duration of the heat treatment are two parameters that could influence the growth process and, by that, the nanoparticle size and aggregation. The technique is nonetheless flexible in the sense that these parameters may be tuned independently. Finally, the large flexibility of the method indicates its applicability also for other types of carbon-supported nanoparticle catalyst materials compatible with sputtering and could be specifically favorable when high demands on precursor purity, surfactant removal, or synthesis environment control are important.

## Conclusions

We have demonstrated a facile synthesis route for supported Pt catalysts, based on a combination of sputtering onto a liquid substrate and a heating step for attachment of Pt particles onto a carbon support. The resulting catalyst size distribution is bimodal with two distinct nanoparticle populations present on the carbon support, where the larger particles (6.7 ± 1.8 nm) dominate over the smaller particles (2.5 ± 0.8 nm) in terms of contribution to the overall catalytic ORR activity. Our results add to the general knowledge of supported catalyst particles produced via sputter deposition in liquids, further establishing the method as a promising candidate for synthesizing this type of catalyst at a large scale. Furthermore, the present method has the potential to be directly extended to other sensitive systems with high demands on precursor purity, surfactant removal, or synthesis environment, indicating the broad applicability of the technique.

## Supporting Information Available

Plots of the kinetic current density of our own polycrystalline Pt disk, Levich analysis of supported Pt nanoparticles, and CO stripping CVs of supported Pt nanoparticles and polycrystalline Pt; complementary TEM images used for particle size distributions and XRD fitting of supported Pt nanoparticles; XPS wide spectrum and Pt 4f narrow scans of supported Pt nanoparticles (PDF)
