# Supplementary material for: Fuel Cell Catalyst Layers with Platinum Nanoparticles Synthesized by Sputtering onto Liquid Substrates
Source: ACS Omega. 2024 Oct 17;9(43):43725–33. doi: 10.1021/acsomega.4c06245 (PMC11525512; doi:10.1021/acsomega.4c06245)
Supplement: Supplementary file 1 — ao4c06245_si_001.pdf [file ao4c06245_si_001.pdf]

Fuel Cell Catalyst Layers with Platinum Nanoparticles Synthesized by Sputtering onto Liquid Substrates

Björn Lönn[1a], Linnéa Strandberg[1a], Vera Roth[1], Mathilde Luneau[2a] and Björn Wickman[1a]*

AUTHOR ADDRESS

[1] Chemical Physics, Department of Physics, Chalmers University of Technology, Gothenburg 412 96, Sweden

[2] Applied Chemistry, Department of Chemistry and Chemical Engineering, Chalmers University of Technology, Gothenburg 412 96, Sweden

[a] Competence Centre for Catalysis, Chalmers University of Technology, 412 96 Gothenburg, Sweden

ORR evaluation on polycrystalline Pt

The kinetic current density of polycrystalline Pt was obtained as explained in the main manuscript and is shown in Figure SI1. A kinetic current density of 1.8 mA/cm^2_{Pt} is observed at 0.9 V vs RHE, which is similar to benchmarks reported in literature[1].

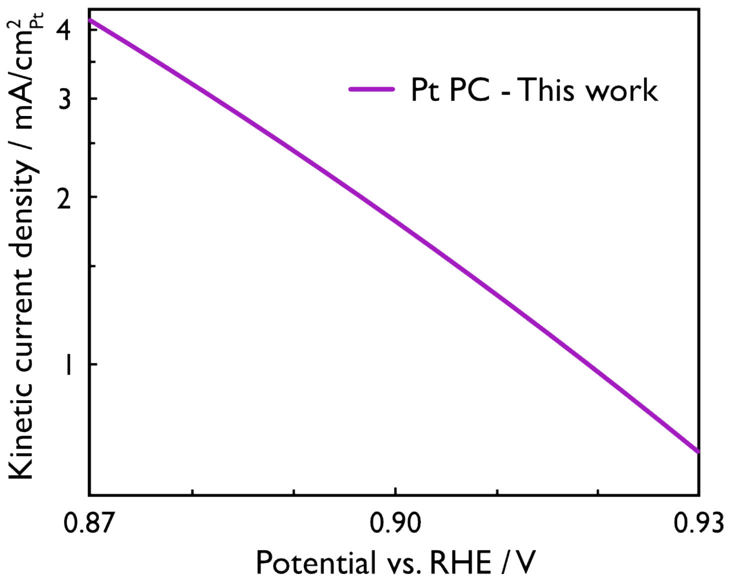

Figure SI1 Kinetic current density plot of polycrystalline Pt (this work).

Electrochemical analysis of Pt/C catalyst

The Levich equation was used to estimate the electron transfer number (*n*) to 3.71, which is close (within 10%) to the theoretical value of 4, assuming the $4e^-$ reaction path for the oxygen reduction.

$$j_L = 0.620 n F D_O^{\frac{2}{3}} \omega^{\frac{1}{2}} \nu^{\frac{-1}{6}} C_O$$

In the Levich equation above, j_L is the Levich current density (A/cm^2), *F* the Faraday constant (96 485 C/mol), D_O the diffusion coefficient of oxygen in 0.1M $HClO_4$ ($1.93 \cdot 10^{-5}$ cm^2/s, from ref. [2]), ω the rotation speed (rad/s), *v* the kinematic viscosity ($1.009 \cdot 10^{-2}$ cm^2/s, from ref. [2]) and C_O the bulk oxygen concentration in the electrolyte ($1.26 \cdot 10^{-6}$ mol/cm^3, from ref. [2]).

Figures SI2 and SI3 depict CO stripping measurements of heat-treated carbon supported Pt nanoparticles and polycrystalline Pt, respectively. The procedure is explained further in the main manuscript, both regarding the measurement details and calculation of the electrochemical surface area (ECSA) for the two different sample types. The resulting ECSAs are shown in Table SI1.

Table SI1 ECSAs of heat-treated carbon-supported Pt nanoparticles and polycrystalline Pt.

**Sample**	**ECSA (cm^2_{Pt})**
Carbon supported Pt nanoparticles on GCE	0.4573
Polycrystalline Pt disk	0.2318

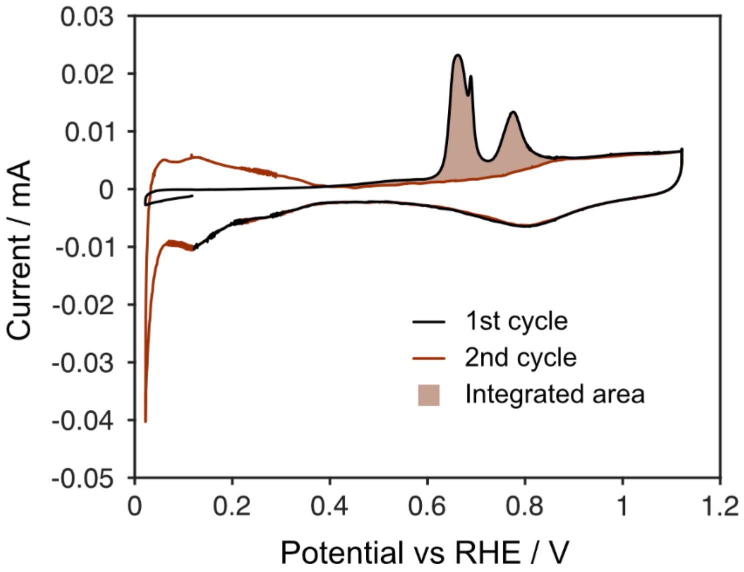

Figure SI2 CO-stripping CVs of Pt NPs. The 1st cycle represents the first CV after poisoning the surface with CO, and the 2nd cycle the subsequent CV after CO oxidation. The filled area represents the integral used for ECSA calculations.

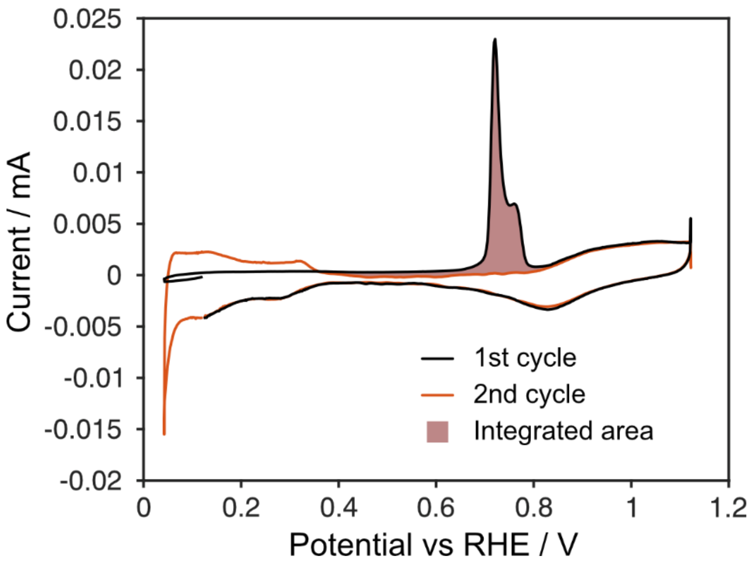

Figure SI3 CO-stripping CV of polycrystalline Pt (this work). The 1st cycle represents the first CV after poisoning the surface with CO, and the 2nd cycle the subsequent CV after CO oxidation. The filled area represents the integral used for ECSA calculations.

Transmission electron microscopy

Complementary TEM images used to retrieve size distributions are shown in this section. Images of as-sputtered particles were obtained using a FEI Tecnai instrument, while the supported catalysts were imaged by a FEI Titan microscope.

As-sputtered primary particles

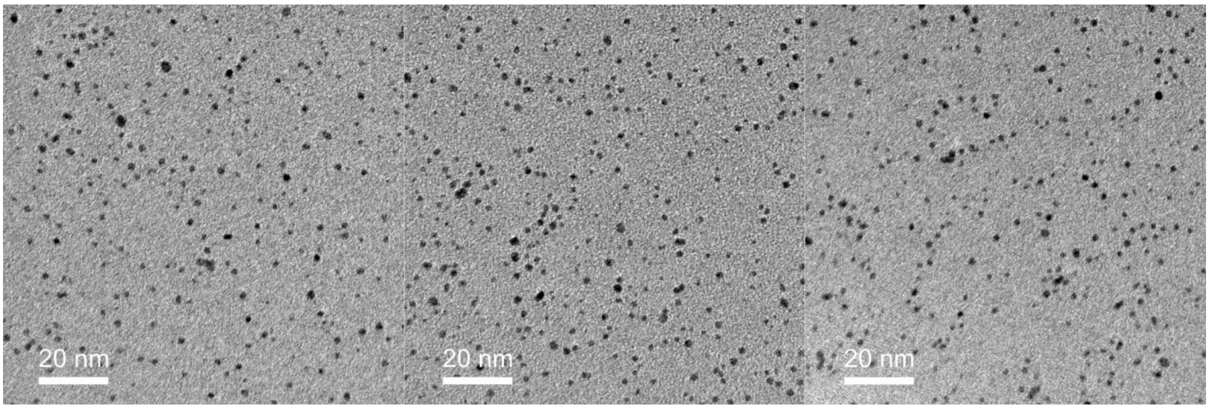

Figure SI4 TEM images used for measuring nanoparticle sizes of as-sputtered primary Pt particles. All scale bars correspond to 20 nm.

Supported nanocatalysts

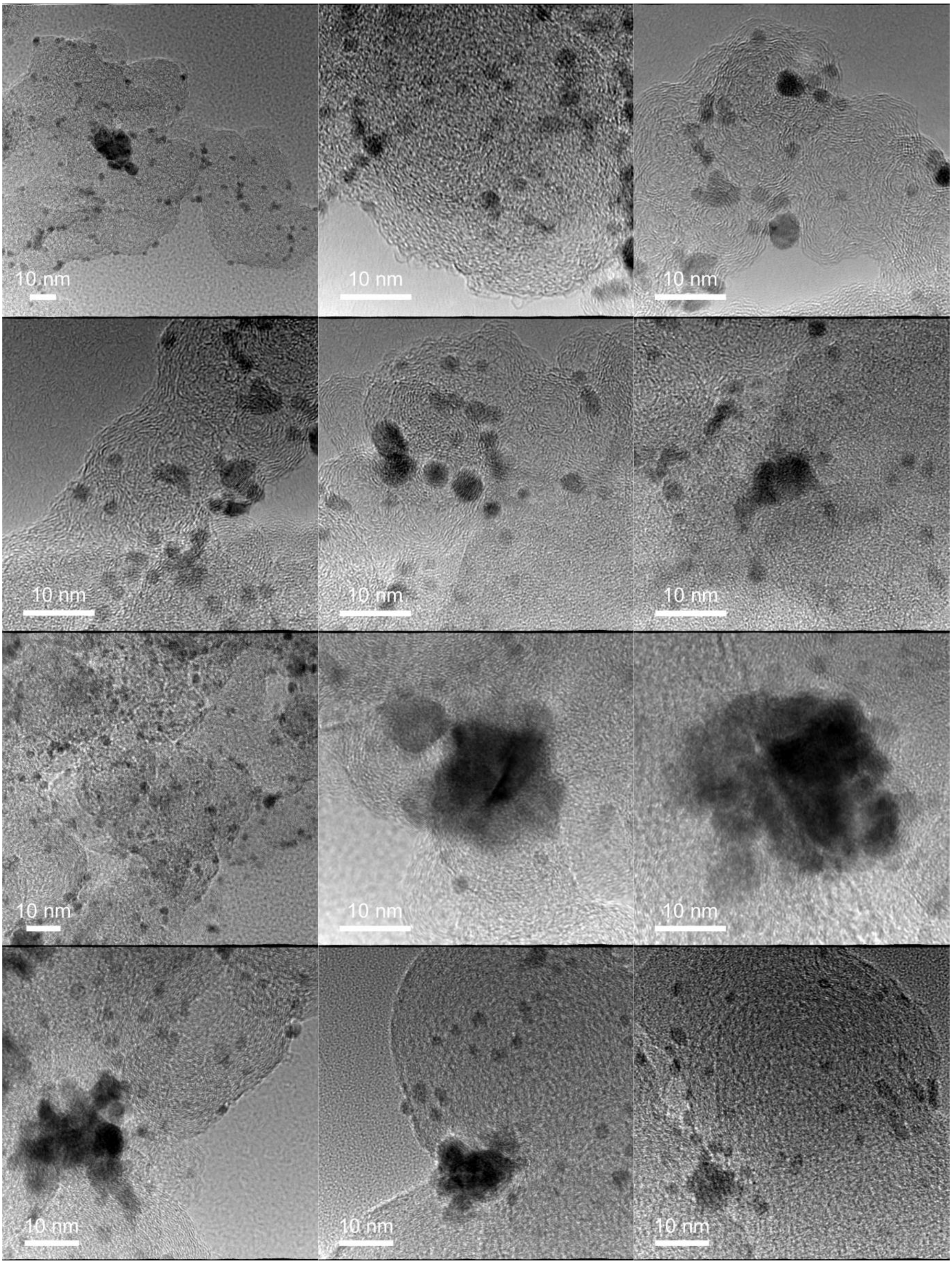

Figure SI5 TEM images used for measuring nanoparticle sizes of supported, heat-treated nanocatalysts. All scale bars correspond to 10 nm.

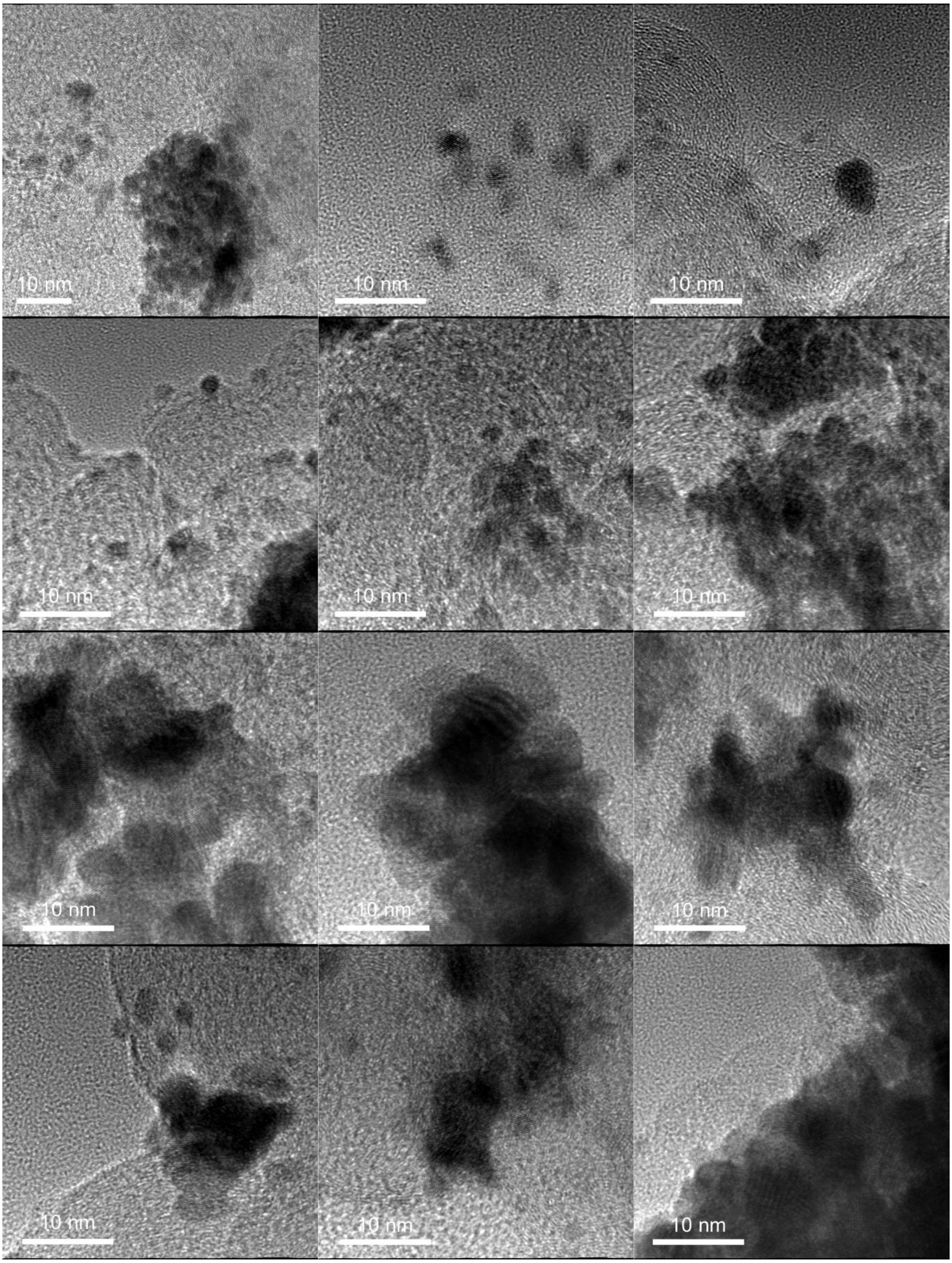

Figure SI6 TEM images used for measuring nanoparticle sizes of supported, heat-treated nanocatalysts. All scale bars correspond to 10 nm.

X-Ray Diffraction

The XRD data and fit are shown in Figure SI7. These measurements were performed to support the TEM size estimations also at the sample bulk level. A good fit with the experimental data was obtained when fitting two Pt phases (see Figure SI7), exhibiting crystallite sizes of 4.6 and 2.4 nm. To obtain the fit, these two phases required the use of two distinct lattice parameters in the fitting, according to Table 2, corresponding to (111) spacings of 2.26 Å and 2.3 Å, respectively. This tensile strain of roughly 2% within the smaller crystallites agrees with our observations in TEM. Furthermore, the 4.6 nm crystallites observed here show that nanoparticle growth far past 3 nm occurs also at the bulk level, during the heat treatment for carbon supported Pt nanoparticles.

Table SI2 XRD fitting details.

**Phase**	**Lattice parameter in fit (Å)**	**Crystallite size (nm)**	**Corresponding (111)-spacing (Å)**	**Phase fraction (%)**
Platinum 1 (larger)	3.92	4.6	2.26	63.85
Platinum 2 (smaller)	3.986	2.4	2.3	36.15

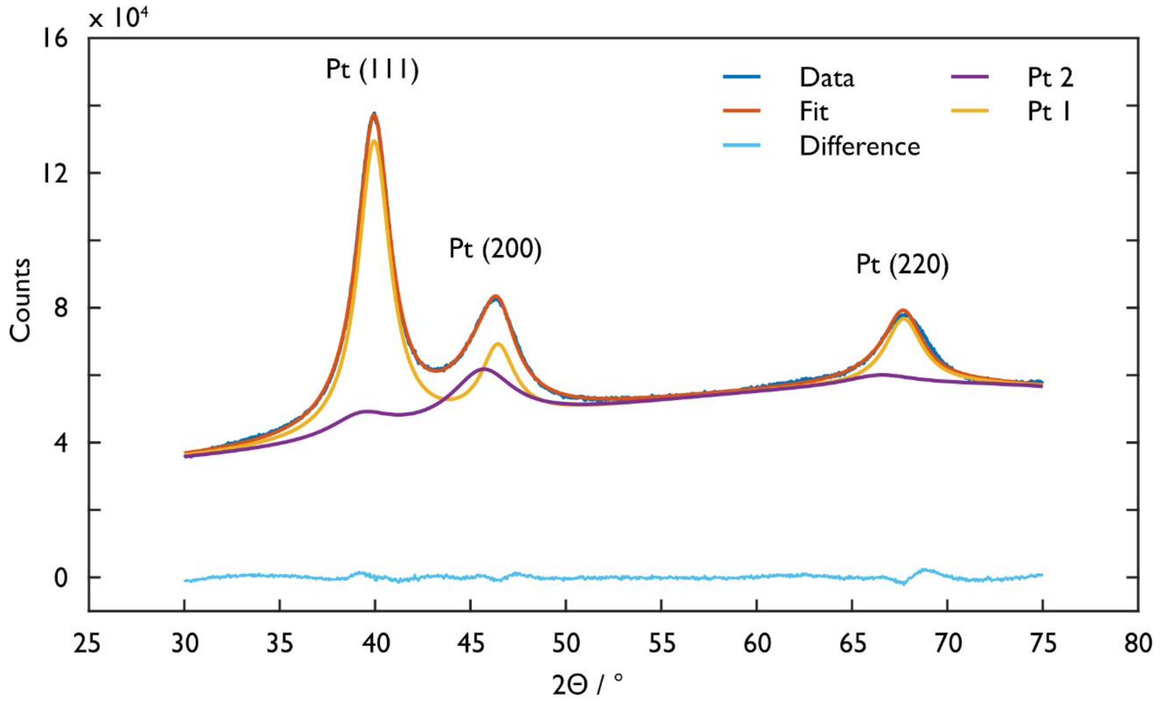

Figure SI7 Experimental XRD pattern (dark blue) of the carbon-supported Pt nanoparticle sample, and the corresponding fit (orange) using two Pt phases (Pt 1 – yellow, and Pt 2 - purple). The background (green) and the difference (light blue) between the fit and data are also displayed.

X-Ray Photoelectron Spectroscopy

XPS wide spectrum of the as-prepared catalyst powder is shown in Figure SI8, displaying clear Pt4d, Pt4f, and Pt5p peaks, along with the 1s peaks of C and O.

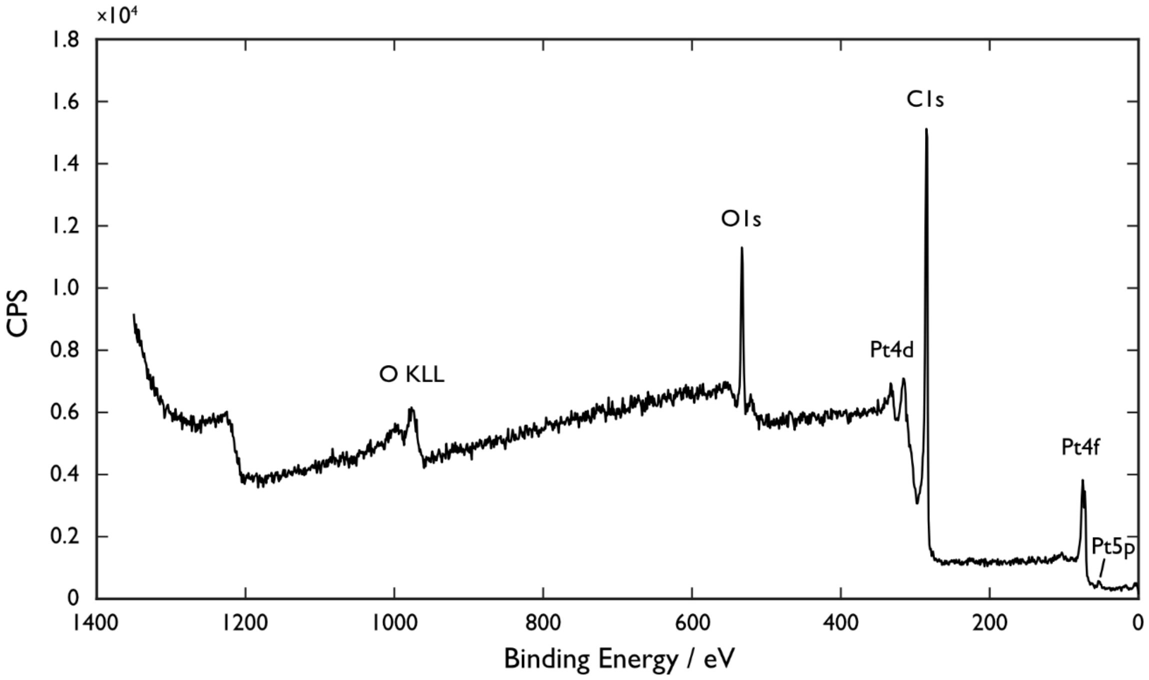

Figure SI8 XPS wide spectrum showcasing the main Pt, C and O peaks.

Narrow scans of the Pt4f doublet peak are displayed in Figure SI9, for Pt/C catalyst powders as prepared and after ORR. The ORR tested sample agrees very well with the reference value for metallic Pt [3] at 71.2 eV and a peak separation of 3.3 eV. In comparison, the as prepared catalyst exhibits a small shoulder situated around 72.5 eV, which indicates a partly oxidized Pt surface, for this sample, possibly in the form of PtO, as the position of the shoulder fits well with that of Pt^{2+} reported by Arrigo et al. [4].

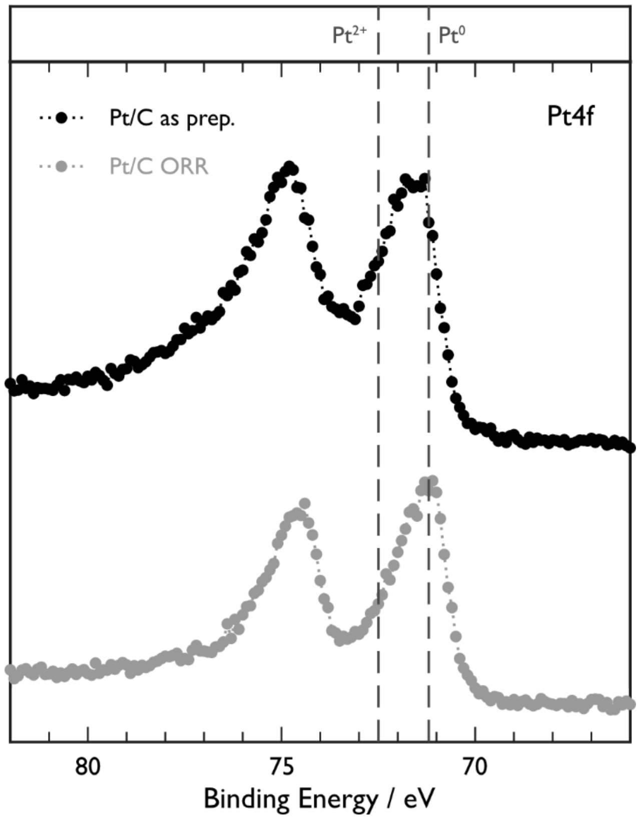

Figure SI9 Pt4f narrow scans of Pt/C catalyst powders as prepared and after ORR. Values for Pt^0 and Pt^{2+} were retrieved from references [3] and [4], respectively.

References

(1) Zamburlini, E.; Jensen, K. D.; Stephens, I. E. L.; Chorkendorff, I.; Escudero-Escribano, M. Benchmarking Pt and Pt-lanthanide sputtered thin films for oxygen electroreduction: fabrication and rotating disk electrode measurements. *Electrochim Acta* **2017**, *247*, 708-721. DOI: 10.1016/j.electacta.2017.06.146.
(2) Markovic, N. M.; Gasteiger, H. A.; Grgur, B. N.; Ross, P. N. Oxygen reduction reaction on Pt(111): effects of bromide. *J Electroanal Chem* **1999**, *467* (1-2), 157-163. DOI: Doi 10.1016/S0022-0728(99)00020-0.
(3) Moulder J.F., S. W. F., Sobol P.E., Bomben. K.D. *Handbook of X-ray Photoelectron Spectroscopy: A Reference Book of Standard Spectra for Identification and Interpretation of XPS Data*; Physical Electronics Division, Perkin-Elmer Corporation, 1992.
(4) Arrigo, R.; Hävecker, M.; Schuster, M. E.; Ranjan, C.; Stotz, E.; Knop-Gericke, A.; Schlögl, R. In Situ Study of the Gas-Phase Electrolysis of Water on Platinum by NAP-XPS. *Angew Chem Int Edit* **2013**, *52* (44), 11660-11664. DOI: 10.1002/anie.201304765.
